# Glucagon Like Peptide 1 and MicroRNA in Metabolic Diseases: Focusing on GLP1 Action on miRNAs

**DOI:** 10.3389/fendo.2018.00719

**Published:** 2018-12-07

**Authors:** Barbara Capuani, Francesca Pacifici, David Della-Morte, Davide Lauro

**Affiliations:** ^1^Department of Systems Medicine, University of Rome “Tor Vergata,”, Rome, Italy; ^2^Department of Medical Science, University Hospital—Fondazione Policlinico di Tor Vergata, Rome, Italy

**Keywords:** GLP1, miRNA, diabetes, lipids, metabolism

## Abstract

Glucagon like peptide 1 (GLP1) is an incretin hormone released from the enteroendocrine L-type cells of the lower gastrointestinal tract. The active isoforms of GLP1 are rapidly degraded (<2 min) by protease dipeptidyl peptidase-4 (DPP-4) after their release. Among its functions, GLP1 exerts a pivotal role in regulating glucose and lipid metabolism. In particular, GLP1 increases glucose stimulated insulin secretion, functional pancreatic β-cell mass and decreases glucagon secretion from pancreatic α-cells. GLP1 can also be a regulator of lipid and lipoprotein metabolism ameliorating diabetic dyslipidemia, liver steatosis, and promoting satiety. Interestingly, it has been found that GLP1 and GLP1 agonists can modulate the expression of different microRNAs (miRNAs), a ~22 nucleotides small non-coding RNAs, key modulators of protein expression. In particular, in pancreas, GLP1 increases the expression levels of miRNA-212 and miRNA-132, stimulating insulin secretion. Similarly, GLP1 decreases miRNA-338 levels, leading to an increase of pancreatic β-cell function, followed by an improvement of diabetic conditions. Moreover, GLP1 modulation of miRNAs expression in the liver regulates hepatic lipid storage. Indeed, GLP1 down-regulates miRNA-34a and miRNA-21 and up-regulates miRNA-200b and miRNA-200c expression in liver, reducing intra hepatic lipid accumulation and liver steatosis. Clinical and pre-clinical studies, discussed in this review, suggest that modulation of GLP1/miRNAs pathway may be a useful and innovative therapeutic strategy for prevention and treatment of metabolic disorders, such as diabetes mellitus and liver steatosis.

## GLP1: An Important Regulator of Metabolic Chronic Diseases

**Figure 1:**
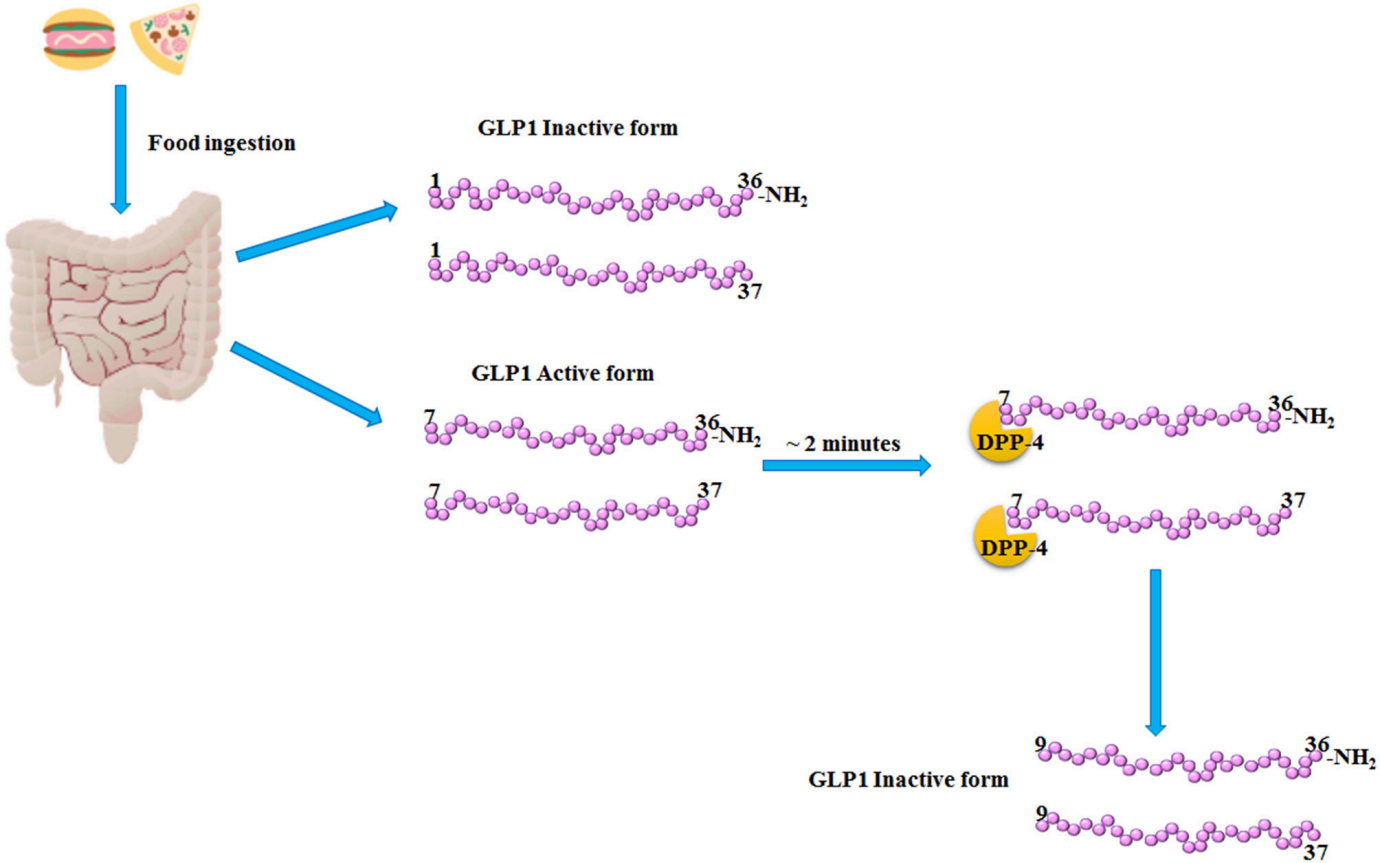
GLP1 release and inactivation. After food ingestion, GLP1 is secreted by the L-cells of lower intestine as GLP1 (1–36) NH_2_ and GLP1 (1–37), which are inactive, and as GLP1 (7–36) NH_2_ and GLP1 (7–37) which are the bioactive forms. Subsequently, within 2–3 min from its release, the dipeptidyl peptidase-4 (DPP-4) cleaved the bioactive forms of GLP1, producing the inactive products, GLP1 (9–36) NH_2_ and (9–37).

Glucagon-Like Peptide 1 (GLP1) is a 30 amino acids incretin hormone mainly produced and secreted by L cells from the lower intestine following meal ingestion. GLP1 secretion becomes very rapid and has two phases: the early phase occurs within 5–15 min after nutrient ingestion, while the late phase arises after 30–60 min, with two different peaks (1). GLP1 is secreted primarily in two forms, GLP1 (7–36), and GLP1 (7–37), which are the bioactive forms with an equipotent insulinotropic effect (1, 2). At position 2 of its aminoacid sequence, GLP1 isoforms (7–36) and (7–37) are cleaved and inactivated by the dipeptidyl peptidase-4 (DPP-4), within <2 min from their release (3) (Figure 1). In order to counteract the activity of DPP-4 increasing the bioavailability of circulating GLP1, DPP-4 inhibitors, also known gliptins, have been developed (4). Moreover, GLP1 receptor agonists (GLP1-RAs) resistant to DPP-4 cleavage have been synthetized (4). Gliptins and GLP1-RAs are recommended as hypoglycemic agents for the treatment of Type 2 Diabetes Mellitus (T2DM).

### GLP1 and Pancreatic Beta Cell

The human GLP1-R (GLP1-receptor) is broadly expressed in all tissues. In particular, GLP1-R is mainly localized in pancreatic β-cells, although its expression in the islet of Langerhans has also been reported in α and δ cells (5, 6). GLP1 enhances glucose-stimulated insulin secretion (GSIS) and reduces glucagon secretion (7, 8). GLP1 stimulation of β-cell lines, in fact, amplifies plasma membrane depolarization subsequent to the closure of K_ATP_ dependent channels, and increases the refilling of the readily-releasable pools of insulin granules (9), through the activation of cAMP-PKA (10). Moreover, GLP1 raise the activity of several voltage dependent calcium channels enhancing calcium intracellular influx, necessary for insulin granules exocytosis (5, 11).

Additionally, GLP1 improves murine pancreatic islets growth (12) and differentiation by activating pancreatic duodenal homeobox-1 (Pdx1) transcription factor (13, 14). All these findings suggest a pleiotropic role of GLP1 on pancreatic beta cells that results in its powerful therapeutic effect.

### GLP1 and Lipid Metabolism

GLP1 decreases lipids absorption by reducing intestinal chylomicron output and postprandial hypertriglyceridemia, which are predictors of cardiovascular risk (4). Treatment with GLP1-RAs or gliptins reduces low density lipoprotein cholesterol (LDL-C), total cholesterol (TC), and triglycerides (TAG) levels in T2DM and obese individuals (15, 16). Although, hematic concentration of high-density lipoprotein cholesterol (HDL-C) was not affected by GLP1-RAs treatment, it has been reported as its function was ameliorated in diet-induced obese rats after Liraglutide (GLP1-RA) administration, which restores HDL-mediated cholesterol efflux capacity (17).

Furthermore, in patients with non-alcoholic fatty liver disease (NAFLD) and non-alcoholic steatohepatitis (NASH), glucose-induced GLP1 secretion was significantly decreased, suggesting that down-regulation of GLP1 levels may contribute to NAFLD and NASH (18). According to these results, several pre-clinical and clinical studies reported that GLP1-RAs and/or gliptins treatment reduced hepatic steatosis and ameliorated hepatic dyslipidemia (19–21). This highlights the potential benefic effect of GLP1 and GLP1-based therapies in NAFLD (18). In agreement with the possible role of GLP1 and GLP1-RAs in counteracting NAFLD, a meta-analysis conducted by Dong and colleagues outlined that Exenatide and Liraglutide improved liver histopathology, and reduced body weight and aminotransferase levels (22). Therefore, based on these evidences, gliptins and GLP1-RAs may be considered potential novel drugs for the treatment of NAFLD.

## miRNAs and Metabolic Disease

MicroRNAs (miRNAs) are ~22 nucleotides non-coding RNA molecules, which act as translational repressor and/or transcript degradation (mRNA) (23, 24). Nowadays, it is expected that more than 45,000 miRNAs target sites are present in human DNA, affecting near 60% of coding genes (25). miRNAs are transcribed as long RNA precursors with a stem-loop structure. Subsequently, are processed into a pre-miRNA, which is a hairpin loop structure of 70 nucleotides. Pre-miRNAs, translocated to cytoplasm where are cleaved into mature miRNAs (26). Mature miRNAs can complementary bind to target mRNA by inducing its degradation or, in absence of complementary association, blocking its translation and resulting in reduced protein synthesis (27). Physiologically, miRNAs regulate several processes, such cell metabolism (28), cell death and division, among others (29).

### miRNAs and Diabetes

Several studies have reported as miRNAs hematic levels may be predictive of the T2DM onset. Indeed, specific miRNAs profile expression has been associated with different pathophysiological state, including Diabetes Mellitus (DM) (30). Many evidences suggest that miRNAs can modulate insulin action and glucose metabolism (31). For instance, miR-126 expression is significantly reduced in both impaired fasting glucose and T2DM individuals (32). Moreover, Yan et al. analyzing miRNAs profile in pre-diabetic and newly diagnosed T2DM subjects, have shown that miR-1249, miR-320b, and miR-527 might be biomarkers of T2DM susceptibility (33). Furthermore, miRNAs can also modulate pancreatic β-cells functionality, suggesting that they can be potential therapeutic targets for DM (34). Recently, a study described as mice knockout for miR-375 displayed a reduced β-cell mass and GSIS, showing hyperglycemia, demonstrating that miR-375 may be an important regulator of pancreatic β-cell functionality (35). Besides these findings, Kloosterman et al. reported a pivotal role of miR-375 in inducing pluripotent stem cells proliferation into pancreatic islets (36). Lastly, changes in the level of miRNAs have been demonstrated to play a role in the development of long-term diabetes complications, such renal fibrosis, visual loss, and lower limb ischemia (37, 38).

### miRNAs and Lipid Metabolism

Evidences linking aberrant miRNAs profile expression with lipid disorders have been demonstrated in clinical and animal experimental models. Among all miRNAs, miR-122 is the most abundantly expressed in liver where it regulates several physiological processes, such TAG and cholesterol metabolism (39). In particular, mice carrying miR-122 deletion, show reduced cholesterol and TAG plasma levels associated to decrease of very low-density lipoprotein (VLDL) formation and release (40). Moreover, the authors reported an accumulation of liver cholesterol and TAG, which contributes to the pathogenesis of liver steatosis (40). According to this result, the silencing of miR-122 significantly increased gene expression of lipid metabolism regulators (41). Taken together, these data suggested that miR-122 plays a pivotal role in NAFLD progression providing a novel potential therapeutic target. However, a controversial study reported as antagonizing miR-122 in mice fed with high fat diet (HFD), showed reduced expression of lipogenic factors and increased lipid oxidation resulting in improved hepatic steatosis (42). The discrepancy with previous studies could arise from the different experimental models employed, and miRNA inhibition system. In particular, Esau et al. (42) used a short-term inhibition of miR-122 with antisense oligonucleotide, while Tsai et al. (40) used a knockout mice model from miR-122 from the germline. It is well-known that miR-122 expression increased in mice liver during embryogenesis probably contributing to liver development and hepatocytes differentiation (43). Thus, impairing miR-122 expression from germline may induce a more negative effect on liver (increased levels of steatosis and fibrosis) and lipid metabolism compared to a short-term inhibition. Moreover, Tsai et al. analyzed the expression levels of microsomal TG transfer protein (MTTP) which is responsible for VLDL assembly and secretion. MTTP levels were downregulating in knockout mice for miR-122 suggesting that MTTP reduction by impairing TG release promotes intrahepatic accumulation contributing to steatosis and fibrosis.

Higher levels of miR-34a were found in patients with NAFLD and in ob/ob mice, which might be involved in the modulation of fatty acid synthesis and oxidation, and in the metabolism of cholesterol and TAG (44). Lately, Ding et al. have found that miR-34a expression was enhanced in human hepatocytes treated with free fatty acids (FFA) and in mice fed with high fat diet (HFD) (45). Inhibition of miR-34a in both models significantly improved steatosis and triglycerides plasma levels, suggesting that miR-34a may be a novel pharmaceutical target for NAFLD (45).

All these evidences suggest that liver and circulating miRNA profiles correlate with liver histological changes and may be used as diagnostic biomarkers for hepatic lipid dysfunction.

## GLP1 and miRNAs in Diabetes

Different cellular signaling pathways have been identified to be activated by GLP1 in pancreatic β-cells. GLP1, mainly, stimulates adenylyl cyclase leading to higher cellular levels of cAMP, followed by the activation of PKA and EPAC and, finally by potentiation of GSIS (5). However, the molecular mechanisms leading to enhanced GSIS in response to GLP1 have not been completely elucidated. Different evidences underlie that GLP1 action on pancreatic β-cells can be mediated by miRNAs profile expression. GLP1, in fact, selectively induces the expression of miR-132 and miR-212, which can increase glucose and GLP1-stimulated insulin secretion (46) (Table 1). These findings were confirmed in freshly isolated rat, mouse and human pancreatic islets after 24 h of GLP1 administration (46). In order to further validate these results, authors treated C57BL/6N mice with Exendin-4 (Ex-4), a GLP1-RAs, and GLP1, observing a dose dependent increase of miR-132 and miR-212 expressions in association with reduced plasma glucose levels (46). Moreover, the increase in miRNAs expression was directly related to cAMP/PKA signaling pathway activation since the inhibition of cAMP dramatically blunted the up-regulation of miR-132 and miR-212 (46). The increase of miR-132 and miR-212 expression was mediated by GLP-1 and remained unexpectedly significant after 8 h and the effect lasted for 24–48 h. High levels of these miRNAs might stimulate GSIS by downregulating carnitine acyl-carnitine translocase (CACT) gene expression and increasing β cell survival (51). Furthermore, obesity increases the expressions of miR-132 and miR-212 in the islets of diabetes-resistant (B6) and diabetes-susceptible (Black and Tan, BRachyury) mice, with higher levels in B6 ~13-fold compared to Black and Tan, BRachyury ~3-fold mice (52), suggesting that these miRNAs may be implicated in the pathogenesis of DM.

**Table 1:** Metabolic effects of GLP1, GLP1-RAs, and DPP-4 inhibitors mediated by miRNAs modulation.

**Molecule used**			**Experimental model**	**Targeted miRNA**	**Metabolic effect**	**References**
**GLP1**	**GLP1-RAs**	**DPP-4 Inhibitors**				
GLP1	Exenatide		Cell line Animal Human	↑miR132 ↑miR-212	↑insulin secretion	(46)
	Exenatide		Animal	↓miR-338	↑Pdx1 expression and insulin secretion	(47)
GLP1			Cell line	↓miR-758	↓cholestrol accumulation	(48)
	Liraglutide		Cell line	↓miR34a ↓miR-21	↓hepatic steatosis	(49)
		Sitagliptin	Cell line Animal Human	↑miR-200b ↑miR-200c	↓TAG ↓intracellular lipid accumulation	(50)

*GLP1, Glucagon like peptide 1; GLP1-RAs, GLP1-Receptor Agonists; DPP-4, dipeptidyl peptidase-4; Pdx, Pancrease/duodenum homeobox protein 1; TAG, triglycerides. The up arrow means increased while the down arrow mean reduced*.

Besides these evidences, Keller et al. found that administration of Ex-4 on isolated rat pancreatic islets significantly reduced miR-375 expression via cAMP/PKA dependent pathway (53), even if this result has not yet been confirmed by another study (46) (Table 1). Taken together, these evidences suggest a novel potential GLP1-mediated molecular pathway to exert insulinotropic effect, providing novel targets for innovative therapeutic strategies against diabetes.

Environmental factors, such endocrine disrupting compounds utilized by industries, contribute to the onset of T2DM (54). Among those, urinary levels of Bisphenol A (BPA) has been associated to alteration of glucose metabolism and diabetes (47). In particular, Wei et al. demonstrated that 8 weeks BPA administration induced peripheral insulin resistance linked to an increase in compensatory insulin secretion from pancreatic β-cells in C57BL/6 mice (47). Besides, they also showed an up-regulation of Pdx1 in freshly isolated pancreatic islets from C57BL/6 mice after 8 weeks BPA administration. In order to investigate the regulators of Pdx1, authors found a significant down-regulation of miR-338 (47). Subsequently, a Luciferase reporter assay was performed to study the putative link between miR-388 and Pdx1, finding that miR-338 directly bound the Pdx1 promoter, decreasing Pdx1 gene expression and protein levels. This suggested that BPA action on insulin secretion was mediated by miR-338-Pdx1 axis (47) (Table 1). According to these findings, the authors also showed that *ex vivo* BPA administration for 4 h on freshly isolated islets, significantly increased Pdx1 levels and decreased miR-388 expression, confirming the early adaptation to insulin resistance induced by BPA administration. Lastly, they found that GLP1-R expression was down-regulated in freshly isolated islets chronically treated with BPA for 48 h in association to the increased levels of miR-388. Moreover, in the same experimental condition, an enhancement of pro-apoptotic genes was also observed, which could at least in part explain the reduced expression of Pdx1 and GLP1-R. Accordingly, Ex-4 administration dramatically reduced miR-338 expression, increasing Pdx1 gene expression levels and insulin secretion, this action might be due to its well-established anti-apoptotic effect. Taken together these findings suggest that effects of BPA are finely controlled by the action of GLP1 on the GLP1-R-miR338-Pdx1 axis (47). Increased levels of Pdx1, in fact, are necessary to compensate impaired insulin secretion induced by BPA treatment.

## GLP1 and miRNAs Expression in Lipid Metabolism

Although different actions of GLP1 and miRNA have been reported to affect lipid metabolism, to date little is known about the effect of GLP1 or GLP1-RAs on lipid metabolism based on their specific modulation of miRNAs expression profile. Recently, it has been reported that GLP1 administration in HepG2 cells reduced the expression of miR-758, decreasing the levels of ATP-binding cassette transporter A1 (ABCA1), with an improvement of cholesterol metabolism (48). ABCA1 reduction blunts cholesterol efflux and HDL-C formation lowering aberrant cholesterol accumulation, associated with defects of lipid metabolism, DM and atherosclerosis (48). These findings suggest that GLP1 may be a new therapeutic target of hypercholesterolemic diseases. A study conducted by Shen on *in vitro* model of liver steatosis, demonstrated as administration of Liraglutide significantly decreased intracellular lipid droplets (49). The miRNAs expression profile in response to FFA and Liraglutide revealed about 123 miRNAs differently expressed. Liraglutide increased miRNAs that were down-regulated by FFA treatment, and those one that were up-regulated by FFA administration were down-regulated by additional treatment with Liraglutide, highlighting the potential miRNAs involvement in Liraglutide-mediated protection against hepatic steatosis (49). Amongst them, miR-34a and miR-21, which are well-known biomarkers of lipid disorders (45), were markedly increased by FFA administration (49). Additional treatment with Liraglutide dramatically reduced miR-34a and miR-21 expression, suggesting that these miRNAs may be therapeutic targets of GLP1-RAs, improving liver steatosis, and supporting the hypothesis to utilize GLP1-RAs for NAFLD treatment (Table 1).

Recently, a study conducted in mice fed with HFD and in human subjects with NAFLD, reported the role of sitagliptin (DPP-4 inhibitor) as modulator of miR-200 family, resulting in an improvement of hepatic steatosis (50). In particular, a significant reduction in miR-200b and miR-200c expression was found, compared to control groups, in association to increased levels of lipogenic factors, such as sterol regulatory element-binding proteins (SREBP1) and Fas (50). These results were, also, confirmed in murine hepatic cell lines, where inhibition of miR-200b and miR-200c resulted in increased TAG levels and enhanced expression of SREBP1 and Fas, suggesting that miR-200 family contributes to abnormal lipid accumulation (50). According to this finding, miR-200b and miR-200c overexpression in murine cell lines and in HFD mice, significantly blunted the intracellular lipids accumulation and reduced SREBP1 and Fas expression (50). Since sitagliptin has been demonstrated to reduce lipid accumulation in rats with diet-induced NAFLD and in T2DM patients (55, 56), the researchers treated murine cell lines and HFD mice with sitagliptin to evaluate its effect on lipid metabolism and miR-200 family (50). Sitagliptin treatment rescued miR-200b and miR-200c expression levels, reduced TAG and intracellular lipid accumulation, decreasing the protein levels of SREBP1 and Fas; these results suggest that sitagliptin effect on liver steatosis may be mediated by miR-200 family (50).

## Conclusion

In this manuscript we reported the state of art on GLP1 mediated miRNAs expression profile modulation and its effect on glucose and lipid metabolism. Furthermore, some specific aspect of GLP1 and miRNAs function in metabolic diseases, mainly T2DM, have been explored. All these findings improved the knowledge of GLP1 action. In particular they shed light on the molecular mechanism by which GLP1 contributes to ameliorate lipid and glucose metabolism throughout miRNAs modulation. Once understood the intracellular pathway, it could be useful to develop novel therapeutic strategies targeting the different miRNAs to counteract a specific pathological aspect (e.g., lipid metabolism rather than glucose alterations) without affecting whole human body and especially avoiding or reducing systemic side effects. Then, we can assert that miRNAs might be considered novel therapeutic targets to treat hyperglycemia and dyslipidemia. In fact, it could be possible to target altered miRNAs expression profile with the aim to restore the defect present in different metabolic pathways to counteract chronic diseases like T2DM and NAFLD.

It might be useful, to explore more in depth which are the differently expressed miRNAs in response to GLP1 (physiological conditions) and GLP1-R agonists (supra-physiological levels), to better understand which miRNAs have a potential impact on metabolic diseases. Moreover, it could be also helpful to consider miRNAs which modulate the GLP1-R expression. In particular, miR-204 has been reported to downregulate GLP1-R expression in β-cells impairing glucose tolerance and insulin secretion (57). Thus, targeting miR-204 could improve β-cells function preventing at least in part the onset of T2DM. Recently, miRNA therapies were applied in oncology pre-clinical and clinical studies, such as the sandwich RNA inhibition; this strategy is realized with multiple different agents acting to major molecular alteration of the disease. Using this approach, the combination of siRNA (small interfering RNA) of EphA2, which inhibits its expression and miR-338 suppressed gastric tumor cells growth (58).

In recent clinical studies, the antimiR against miR-103/107 were used for the treatment of NASH, in affected patients and obese mice. These anti-miRNAs silencing miR-103/107 improved insulin sensitivity mediated by caveolin-1, ameliorating liver function (59). Moreover, it has been recently reported that several miRNAs are dysregulated in diabetes and their restoration improves metabolic dysfunctions and the severity of its complications (60). Since to date, the gliptins and GLP1-RAs are important drugs for the treatment of T2DM, it could be of interest to find and target “new” specific miRNAs substrates modulated by GLP1, which affect the pathogenesis of metabolic diseases.

Finally, new data about the use of miRNAs as a drug in pre-clinical studies and additional molecular information regarding GLP1-mediated miRNAs regulation may generate a useful strategy in the innovative field of miRNAs therapy to treat metabolic diseases.

## Author Contributions

BC identified the topic of the review and wrote paper. FP prepared figures and wrote paper. DD-M revised the literature and the manuscript. DL wrote and revised the paper.

### Conflict of Interest Statement

The authors declare that the research was conducted in the absence of any commercial or financial relationships that could be construed as a potential conflict of interest.
